# Single-nucleus analysis reveals microenvironment-specific neuron and glial cell enrichment in Alzheimer’s disease

**DOI:** 10.1186/s12864-024-10447-3

**Published:** 2024-05-28

**Authors:** Jieqiong Xie, Yating Lan, Cuihua Zou, Jingfeng He, Qi Huang, Jingyi Zeng, Mika Pan, Yujia Mei, Jiefeng Luo, Donghua Zou

**Affiliations:** 1 Department of Neurology, The Second Affiliated Hospital of Guangxi Medical University, Nanning, Guangxi 530007 People’s Republic of China; 2 Department of Quality Control, Guangxi Medical University Cancer Hospital, Nanning, Guangxi 530021 People’s Republic of China

**Keywords:** Alzheimer’s disease, Microenvironment, Single-nucleus sequencing, lncRNA-SNHG14, MRTFA, MRTFB

## Abstract

**Background:**

Alzheimer’s disease (AD) is a complicated neurodegenerative disease. Neuron-glial cell interactions are an important but not fully understood process in the progression of AD. We used bioinformatic methods to analyze single-nucleus RNA sequencing (snRNA-seq) data to investigate the cellular and molecular biological processes of AD.

**Method:**

snRNA-seq data were downloaded from Gene Expression Omnibus (GEO) datasets and reprocessed to identify 240,804 single nuclei from healthy controls and patients with AD. The cellular composition of AD was further explored using Uniform Manifold Approximation and Projection (UMAP). Enrichment analysis for the functions of the DEGs was conducted and cell development trajectory analyses were used to reveal underlying cell fate decisions. iTALK was performed to identify ligand-receptor pairs among various cell types in the pathological ecological microenvironment of AD.

**Results:**

Six cell types and multiple subclusters were identified based on the snRNA-seq data. A subcluster of neuron and glial cells co-expressing lncRNA-SNHG14, myocardin-related transcription factor A (MRTFA), and MRTFB was found to be more abundant in the AD group. This subcluster was enriched in mitogen-activated protein kinase (MAPK)-, immune-, and apoptosis-related pathways. Through molecular docking, we found that lncRNA-SNHG14 may bind MRTFA and MRTFB, resulting in an interaction between neurons and glial cells.

**Conclusions:**

The findings of this study describe a regulatory relationship between lncRNA-SNHG14, MRTFA, and MRTFB in the six main cell types of AD. This relationship may contribute to microenvironment remodeling in AD and provide a theoretical basis for a more in-depth analysis of AD.

**Supplementary Information:**

The online version contains supplementary material available at 10.1186/s12864-024-10447-3.

## Introduction

Alzheimer’s disease (AD) accounts for more than 50% of all dementia cases worldwide. The primary clinical symptoms include memory loss and severe disability [1]. Its main pathological features are neurofibrillary tangles (NFTs) and amyloid-β (Aβ) -dominated plaques in the brain of senile patients [2]. The rapidly expanding elderly population within China has led to a corresponding increase in the number of patients with AD. It is projected that by 2050, the number of patients with AD in China will exceed 40 million [3]. However, currently, no standardized drugs or measures are available to effectively delay the progression of AD [4].

Recently, research has highlighted non-neuronal central nervous system (CNS) cells, namely glial cells, as active contributors to AD pathophysiology [5]. Alterations in the communication between neurons and glial cells have been linked to AD [6]. Interactions between microglia and neurons are mediated by intercellular signaling pathways, including purinergic signaling, cytokines, neurotransmitters, and neuropeptides [7]. In addition, a new subcluster of microglia-disease-associated microglia (DAM) was discovered in a mouse model of AD [8]. DAM usually appears under conditions of accumulation of neuronal apoptotic bodies and myelin debris in AD [9]. Microglia play a crucial role in the pathogenesis of AD by facilitating communication through the ligand-receptor axis [10] Astrocytes are central players in coordinating brain homeostasis, and multiple studies have identified specific signatures of astrocytes altered in AD [11–13]. FTH1 and SAT1 were shown to impact astrocyte ferroptosis [14]. Oligodendrocytes also play a crucial role in the pathology of AD due to their roles in producing myelin and supporting axons [15]. In one study, researchers identified an oligodendrocyte subpopulation linked to a disease that emerges as an AD-like pathology in male AppNL-G-F and male 5xFAD AD mouse brains, as well as in postmortem AD human brains; this subpopulation was associated with Erk1/2 signaling dysfunction [16]. Neuron-derived signaling molecules regulate the proliferation, differentiation, and survival of oligodendrocytes. In turn, signals from oligodendrocytes to neurons direct the assembly of specific subdomains in neurons at the node of Ranvier [17]. Therefore, further elucidation of which cell types communicate with one another and how they communicate may help us better understand how AD occurs and progresses. This knowledge may then guide the development of novel diagnostic and therapeutic strategies. Accordingly, the relationships between neurons and glia in AD warrant further investigation.

The rapid advancement of next-generation sequencing (NGS) technologies has enabled new perspectives on intricate biological processes [18]. Single-nucleus RNA sequencing (snRNA-seq), for example, provides opportunities to analyze cellular heterogeneity. It allows researchers to follow the developmental paths of several cell lineages and identify regulatory links between genes [19]. This technology has become a potent tool for comprehending the complexity and dynamics of cells in the central nervous system [20]. In this study, we investigated the cellular environment in AD using single-nucleus sequencing. Our analysis provides an overview of the AD ecosystem and identifies relevant cell clusters. Different from previous studies, we also examine the developmental trajectories, intercellular communication and signaling pathways of these AD cells. Ultimately, we identify specific biomarkers that contribute to AD homeostasis and explore interactions between the different cell types. Our findings provide a deeper understanding of the cellular functions and key signaling pathways involved in AD.

## Materials and methods

### Data sources

To investigate transcriptome changes in different cell types during AD, we obtained the GSE157827 and GSE174367 datasets from the Gene Expression Omnibus database (www.ncbi.nlm.nih.gov/geo) [21]. GSE157827 includes snRNA-seq data from prefrontal cortical samples of 12 patients with AD (8 males and 4 females) averaging 74.6 years of age and 9 normal control subjects (6 males and 3 females) averaging 85.4 years of age, based on the GPL24676 platform [22]. The GSE174367 dataset includes gene-chip data from prefrontal cortical samples of 19 subjects, including 11 patients with AD (5 males and 6 females) and 8 healthy controls (5 males and 3 females), aged between 74 years and above, based on the GPL24676 platform too [23].

### Data preprocessing and construction of single nucleus plot

The single-nucleus sequences from 40 samples resulted in the retention of 24,0804 single nuclei after data processing, including quality control, data filtering, and normalization. Quality control was conducted on single-nucleus seurat objects to assess double cell counts, dead cells, and mitochondrial gene expression. The sample tissues consisted of active brain cells, which exhibit higher mitochondrial content compared to normal cells. Cells and double cells are filtered out based on mitochondrial gene content and RNA amount, respectively. The default filtering thresholds were set at 1% above and below the number of features (genes) and cells with mitochondrial content exceeding 10%. The IntegrateData function [24] was used to merge snRNA-seq data and perform cell clustering analysis with default parameters. We utilized the Seurat package and Unified Manifold Approximation and Projection (UMAP) algorithm for dimensionality reduction and visualization [25] and mapping to a single nucleus. Utilizing the marker genes that have been identified, along with markers that have been validated in past single-cell research and in experimental settings, various cell clusters were then classified into recognized cell types. We identified additional different expression genes using the Wilcoxon rank-sum test with the FindAllMarkers function and parameters logfc.threshold = 0.25 and test.use = wilcox, which had large expression differences between pct.1 and pct.2, as well as changes in multiple markers. These genes exhibited high expression levels in a particular cell type and minimal to no expression in other cell types, making them useful for identifying and characterizing specific cell populations. Then we assigned a cell-type identity to each cell cluster according to the expression of known cell-type markers, according to the location of the single cell map and the correlation of each cluster. Excitatory neurons (ExNeu), inhibitory neurons (InNeu), microglia (Mic), oligodendrocytes (Oli), astrocytes (Ast), and oligodendrocyte progenitor cells (Opc) were reintegrated and aggregated using the Seurat package. The second clustering followed the same process as the first clustering. Additionally, annotation and reclustering were performed based on functional genes [22, 23].

### Differential expression analysis and functional enrichment analysis

Differential expression analysis was conducted using the FindAllMarkers function of the Seurat package [25]. differentially expressed genes (DEGs) were identified in different clusters. DEseq2 was also used to perform differential expressed gene analysis (Supplemental Data Table S1). Due to significant differences in the number of cells between cell subpopulations, the number of differentially expressed genes obtained from DEseq2 is not suitable for subsequent analysis. Therefore, the results obtained from FindAllMarkers analysis were ultimately chosen for further analysis. When the p-value < 0.05, a pathway is considered to be significantly associated with the marker gene. The Benjamin-Hochberg method can be used to correct the p-value and control the false/true positive ratio within a certain range [26]. The enrichment analysis in Gene Ontology (GO) and Kyoto Encyclopedia of Genes and Genomes (KEGG) pathways was determined based on the differential expression gene of cell clusters. The ClusterProfiler package [27] was utilized for this analysis, with *p* < 0.05 deemed significant.

### Single-cell trajectory analysis

Pseudo-time analysis is a useful tool for understanding the dynamics and temporal trajectories of gene expression within cell types and inferring cellular evolution during AD [28]. Using Monocle3, a branch trajectory was constructed to explore the development and differentiation trajectories of a single-cell map of patients [29]. This trajectory was used to simulate the evolutionary trajectory of cellular development in AD pathogenesis and to project the cells into a low-dimensional space through UMAP using Monocle3 default parameters.

### Analysis of intercellular communication

iTalk serves as a publicly available repository of potential receptor-ligand interactions. We utilized it from the R package to analyze the communication between neuron and glial cell subclusters [30] and cytokines, growth factors, immune checkpoints. To identify receptor-ligand interactions, we referred to the STRING database of protein-protein interactions [31].

### Molecular docking of ligands and receptors

To evaluate binding between molecules, the sequences that exhibited the strongest binding ability with proteins were screened (http://s.tartaglialab.com/page/catrapid). After constructing the model, Hex 8.0.0 software was used to dock the constructed model and protein model [32]. Finally, the docking model was visualized using PyMOL software [33].

### Statistical analysis

Statistical analyses were performed using R (https://www.r-project.org/), and gene expression levels were evaluated using an unpaired *t*-test. The significance level was set at *p* < 0.05. A bioinformatics cloud platform was used for analysis (http://www.bioinforcloud.org.cn).

## Results

### Single-nucleus transcriptomic landscape in patients with AD and healthy brain tissue

**Fig. 1:**
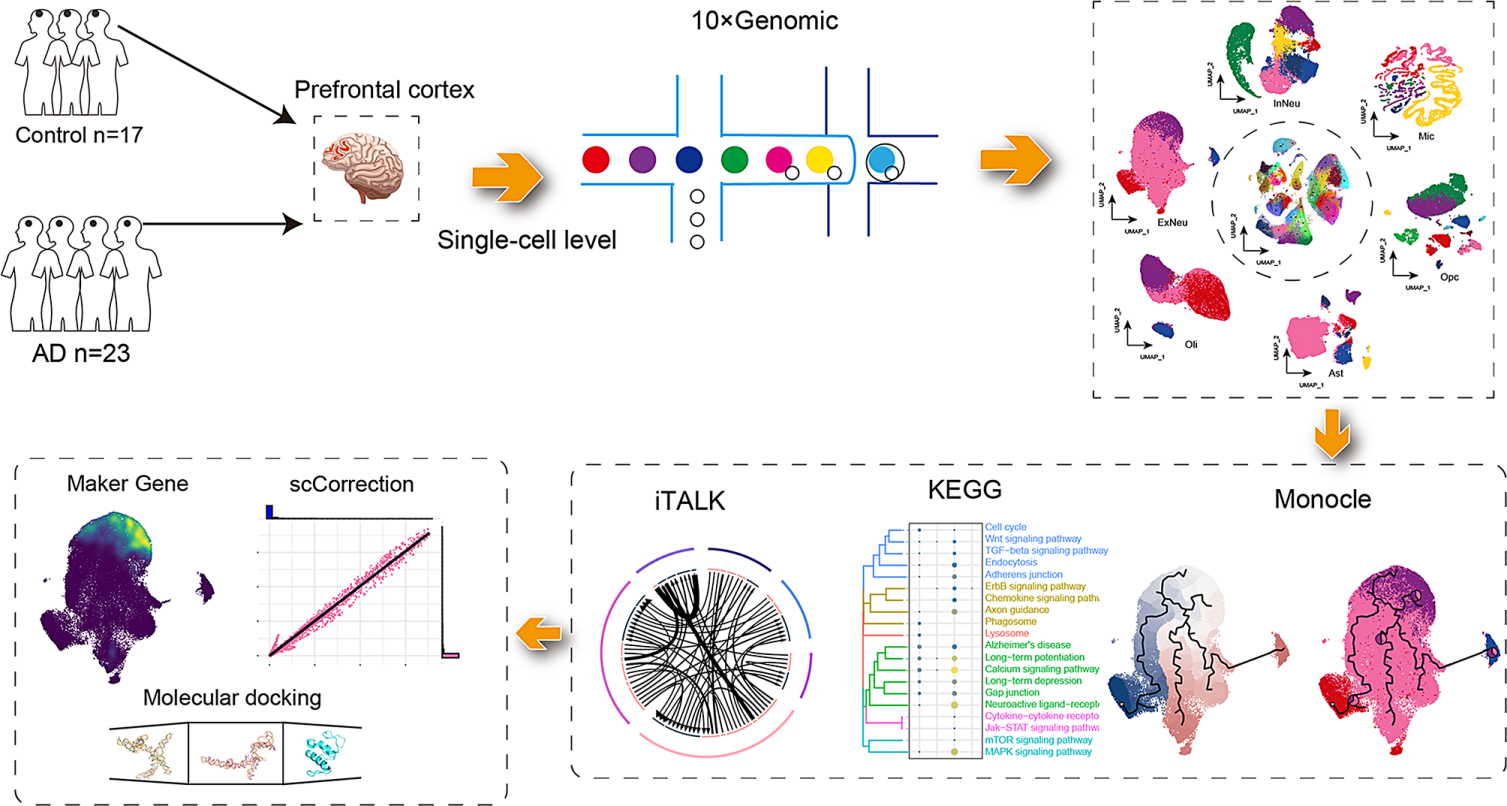
Study design flow chart

**Fig. 2:**
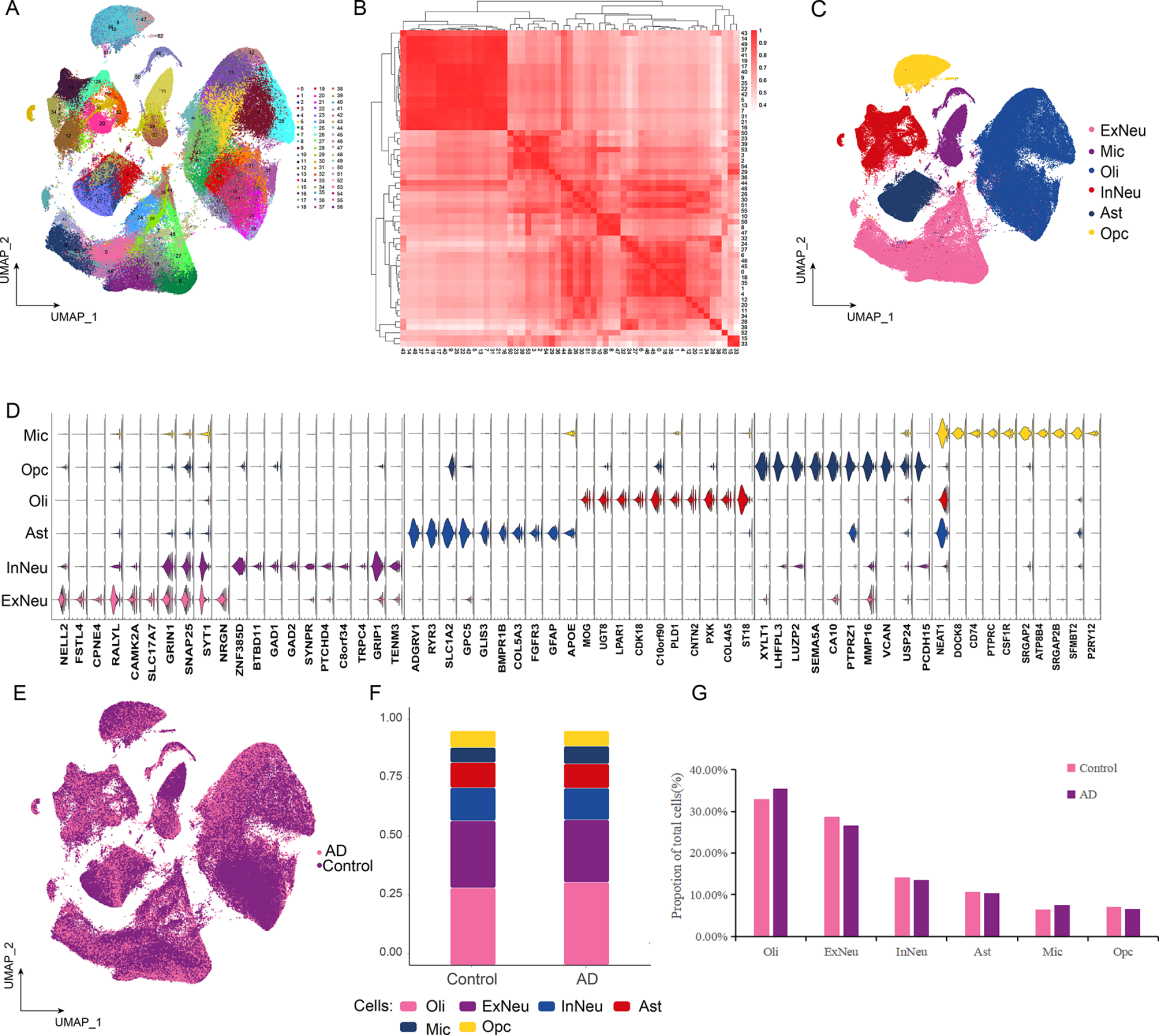
Global single-cell landscape of Alzheimer’s disease (AD). **(A)** The global single-nucleus map identified 57 cell clusters. **(B)** Analysis of correlations between samples. **(C)** AD mapping single-nucleus types: excitatory neuron (ExNeu), inhibitory neuron (InNeu), microglia (Mic), oligodendrocyte (Oli), astrocyte (Ast), and oligodendrocyte progenitor (Opc). **(D)** Violin diagram showing the top ten characteristic genes in different cell types. UMAP, uniform manifold approximation and projection. **(E)** Map of AD and control group distribution in samples. **(F)** Differences in cell abundance components between patients with AD and controls. **(G)** Proportion of total cell numbers in six cell types of patients with AD and controls

To explore the cellular landscape of AD, we analyzed snRNA-seq datasets (GSE157827 and GSE174367) obtained from the GEO database. The workflow of the study is presented in Fig. 1. Clinical information associated with all samples is shown in Supplemental Data Table S2. The data were processed for quality control and normalization (Supplemental Data Figure S1). A total of 24,0804 individual cells were identified, which could be divided into 57 distinct clusters (Fig. 2A). Furthermore, a correlation was detected between the clusters. Based on the expression patterns of different clusters, we further calculated the correlation between them. (Fig. 2B). We recognized a cell type for each cell cluster according to the expression of known cell-type markers [22, 23] and identified additional cell-type specific marker genes using the FindAllMarkers function in R. The 57 clusters were annoted six different cell types: excitatory neurons (ExNeu) (CAMK2A), inhibitory neurons (InNeu) (GAD1, LHFPL3), microglia (Mic) (DOCK8, CSF1R), oligodendrocytes (Oli) (MBP, PLP1, ST18), astrocytes (Ast) (AQP4, ADGRV1, GPC5, RYR3), and oligodendrocyte progenitor cells (Opc) (OLIG1, OLIG2) (Fig. 2C and D, Supplemental Data Table S3). Each cell type was derived from both the control and AD groups (Fig. 2E, Supplemental Data Figure S2). We then analyzed the abundance of cell types and found that they varied similarly in the two groups (Fig. 2F, G). This suggests that AD may be the cause of abnormal gene regulation, and each cell may have its unique expression pattern.

### Functional characterization of excitatory neuron subclusters

**Fig. 3:**
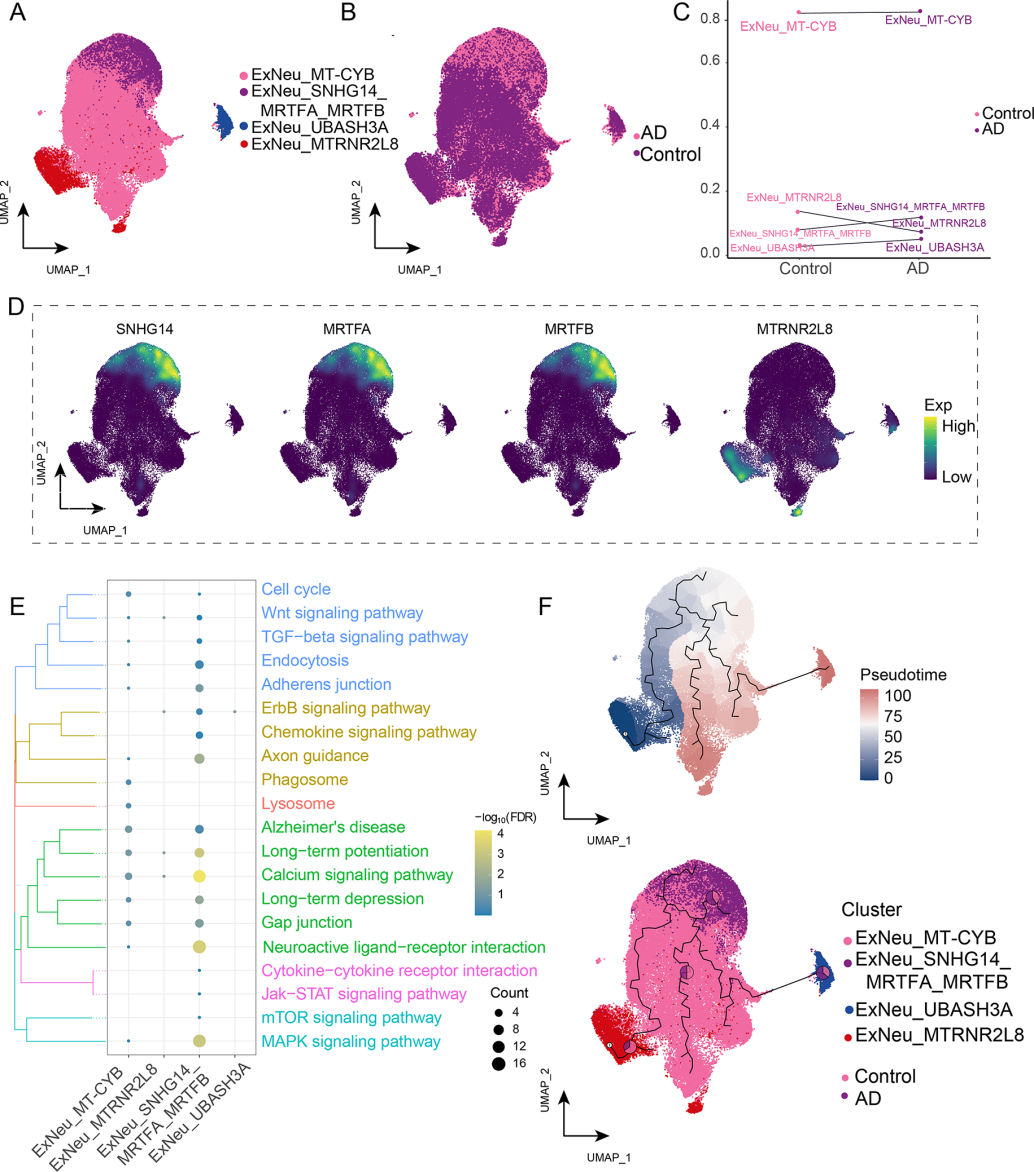
Identification of ExNeu clusters of excitatory neurons (ExNeu) in Alzheimer’s disease (AD). **(A)** Single-nucleus map showing ExNeu cell clusters. **(B)** Single-nucleus map showing the AD group and control group in ExNeu clusters. **(C)** Differences in cell abundance between patients with AD and controls. **(D)** Mapping of marker genes *lncRNA-SNHG14, MRTFA, MRTFB*, and *MTRNR2L8* in ExNeu subclusters. **(E)** Biological pathways of the ExNeu subclusters. The darker the color, the more significant the enrichment. **(F)** Single-nucleus mapping of ExNeu progression and pseudo-time values. The pie chart shows the proportion of different groups in the cluster. ExNeu, excitatory neuron; UMAP, uniform manifold approximation and projection

To evaluate cellular heterogeneity, our analysis of ExNeu identified four cell subclusters based on their characteristic genes (Fig. 3A). All subclusters were present in the AD group (Fig. 3B). We found an increase in the abundance of the ExNeu_SNHG14_MRTFA_MRTFB subcluster and a decrease in the abundance of the ExNeu*_*MTRNR2L8 subcluster in AD (Fig. 3C). This indicates that lncRNA-SNHG14, MRTFA, and MRTFB are highly enriched in ExNeu, whereas MTRNR2L8 expression is down-regulated in cell type. Moreover, lncRNA-SNHG14, myocardin-related transcription factor A (MRTFA), MRTFB, and MT-RNR2-like 8 (MTRNR2L8) were identified in a single-nucleus plot (Fig. 3D), with lncRNA-SNHG14 co-expressed with MRTFA and MRTFB, indicating its potential role in AD. Enrichment analysis revealed that the abnormally regulated genes were associated with neuroactive ligand-receptor interactions, calcium and mitogen-activated protein kinase (MAPK) signaling pathways (Fig. 3E, Supplemental Data Table S4) These pathways were previously found to be associated with AD [34–37]. Hence, the presence of this subcluster may worsen AD features and cause neuronal damage via these pathways. Additionally, pseudo-time analysis showed the cellular development of ExNeu from control to AD (Fig. 3F), indicating that ExNeu_MTRNR2L8 was abundant in the early stage of ExNeu developmental trajectory, while the ExNeu*_*SNHG14*_*MRTFA*_*MRTFB subcluster was prevalent in the late stage of ExNeu developmental trajectory. This suggests that different subclusters may play varying roles in AD development.

### Identification of an SNHG14-positive subcluster in AD inhibitory neurons

**Fig. 4:**
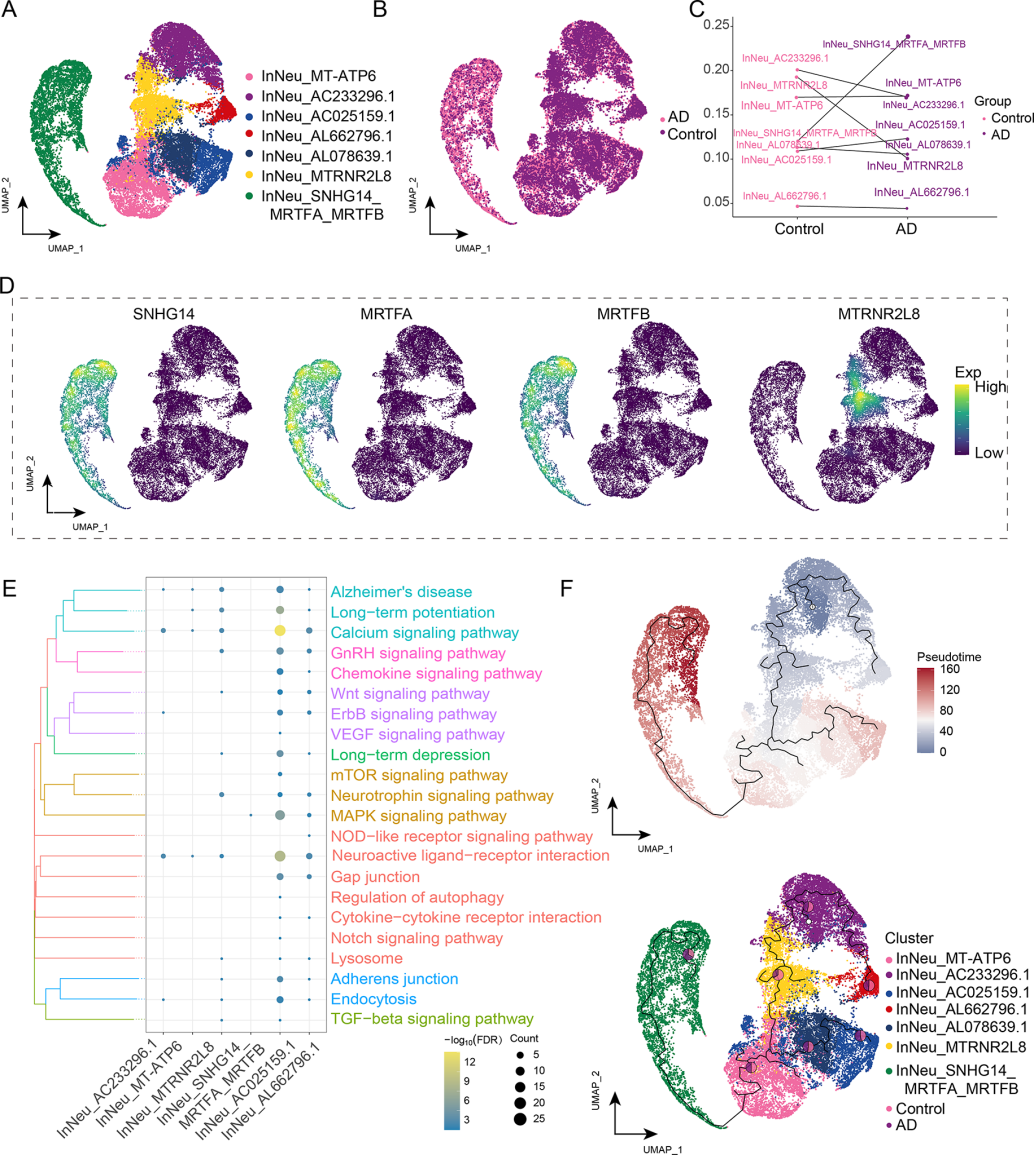
Identification of inhibitory neuron (InNeu) clusters in Alzheimer’s disease (AD). **(A)** Single-nucleus map showing InNeu cell clusters. **(B)** Single-nucleus map showing the AD group and control group in the InNeu cluster. **(C)** Differences in cell abundance between patients with AD and controls. **(D)** Mapping of marker genes *lncRNA-SNHG14, MRTFA, MRTFB*, and *MTRNR2L8* in the InNeu subcluster. **(E)** Biological pathways of the InNeu subclusters. The darker the color, the more significant the enrichment. **(F)** Single-nucleus mapping of InNeu progression and pseudo-time values. The pie chart shows the proportion of different groups in a subcluster. InNeu, inhibitory neuron; UMAP, uniform manifold approximation and projection

We performed a clustering analysis of inhibitory neurons and identified seven distinct subclusters (Fig. 4A) present in AD and control groups (Fig. 4B). However, the abundance of InNeu_MTRNR2L8 decreased and that of InNeu_SNHG14_MRTFA_MRTFB increased in AD. The latter subcluster was the most abundantly increased in patients with AD (Fig. 4C).

Subsequently, we mapped lncRNA-SNHG14, MRTFA, MRTFB, and MTRNR2L8 to a single-nucleus plot and found that SNHG14, MRTFA, and MRTFB exhibited co-expression (Fig. 4D). Enrichment analysis showed that InNeu_SNHG14_MRTFA_MRTFB was significantly enriched in neuroactive ligand-receptor interactions, calcium signaling pathways, and MAPK signaling pathways (Fig. 4E, Supplemental Data Table S5).Pseudo-time analysis (Fig. 4F) further revealed that InNeu_MTRNR2L8 was abundant during the initiation of InNeu developmental trajectory, while InNeu_SNHG14_MRTFA_MRTFB was prominent at the late stage of InNeu developmental trajectory, similar to ExNeu. These subclusters may contribute to neuronal damage and degeneration in AD.

### Overactivation of a microglia cluster promotes AD

**Fig. 5:**
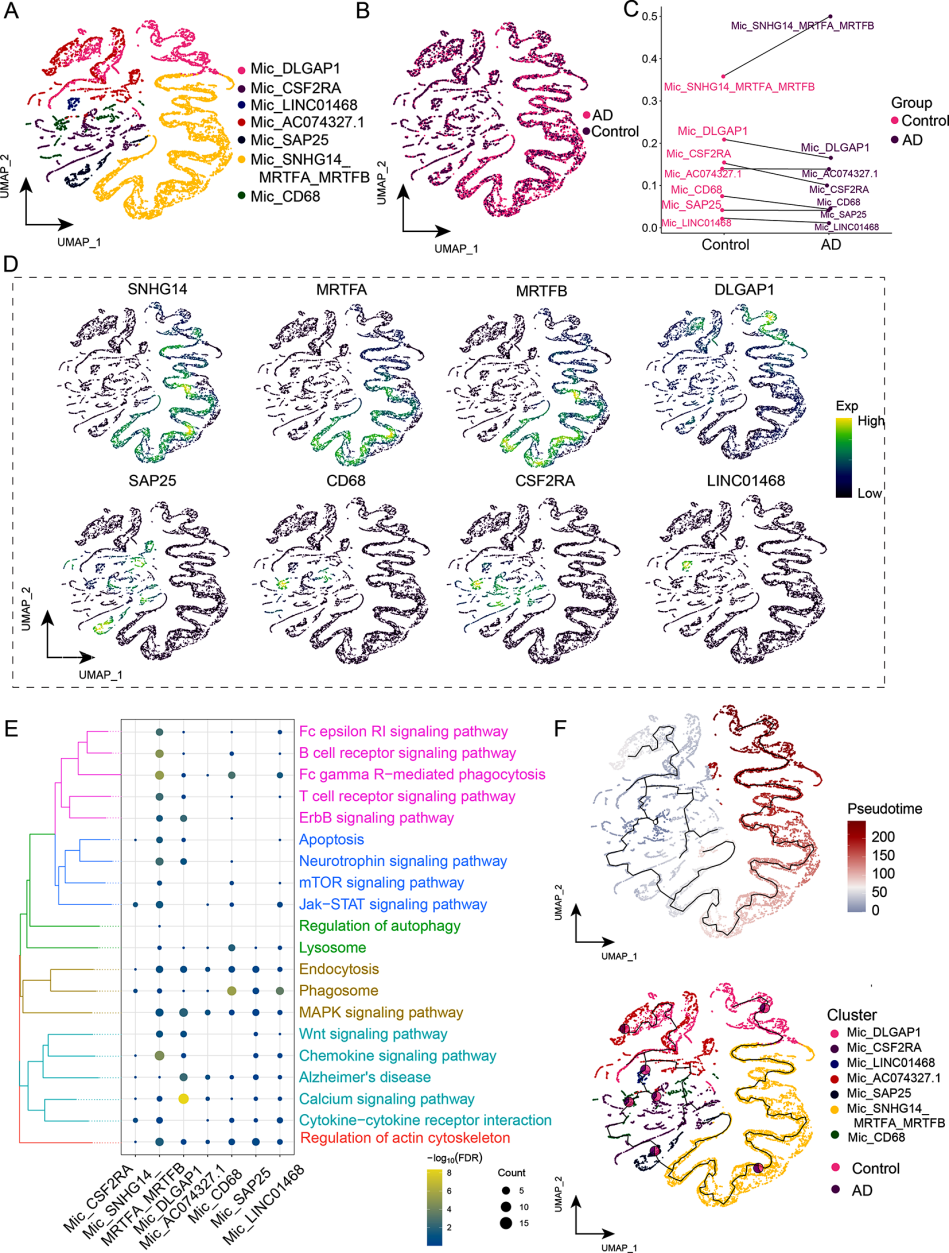
Identification of microglia (Mic) clusters in Alzheimer’s disease (AD). **(A)** Single-nucleus map of the Mic cell cluster. **(B)** Single-nucleus plot of the AD group and control group. **(C)** Differences in the cellular ecological components of Mic subclusters in different populations. **(D)** Marker genes of the Mic subclusters. **(E)** Biological pathways in the Mic subclusters. **(F)** A single-nucleus map was used to map the developmental track of Mic in AD. The pie chart shows the proportion of different groups in a subcluster. Mic, microglia; UMAP, uniform manifold approximation and projection

By reclustering microglia, we identified seven distinct cell subclusters (Fig. 5A), most of which were present in AD groups (Fig. 5B). The abundance of Mic*_*SNHG14*_*MRTFA*_*MRTFB increased, while the other subclusters decreased in the AD group (Fig. 5C). A single-nucleus plot was used to map the expression of marker genes, including *SNHG14, MRTFA, MRTFB, DLGAP1, SAP25, CD68, CSF2RA*, and *LINC01468* (Fig. 5D). lncRNA-SHNG14, MRTFA, and MRTFB were co-expressed in the same cluster. The DEGs in Mic*_*SNHG14*_*MRTFA*_*MRTFB were significantly enriched in immune pathways, including chemokines, Fc gamma R-mediated phagocytosis, B cell receptor and T cell receptor signaling pathways (Fig. 5E, Supplemental Data Table S6) Pseudo-time analysis showed that Mic*_*SNHG14*_*MRTFA*_*MRTFB was abundant during the late stage of Mic developmental trajectory. (Fig. 5F). A recent study showed that chemokines secreted by microglia may contribute to synaptic plasticity and cognitive impairment associated with AD [38]. Moreover, during the late stages of AD, excessive activation of cells triggers an exaggerated inflammatory response, which worsens the neurodegenerative process [39]. Therefore, this subcluster may be responsible for the overactivation of microglia, ultimately leading to increased AD severity.

### An astrocyte subcluster is associated with apoptosis-related signaling pathways in AD

**Fig. 6:**
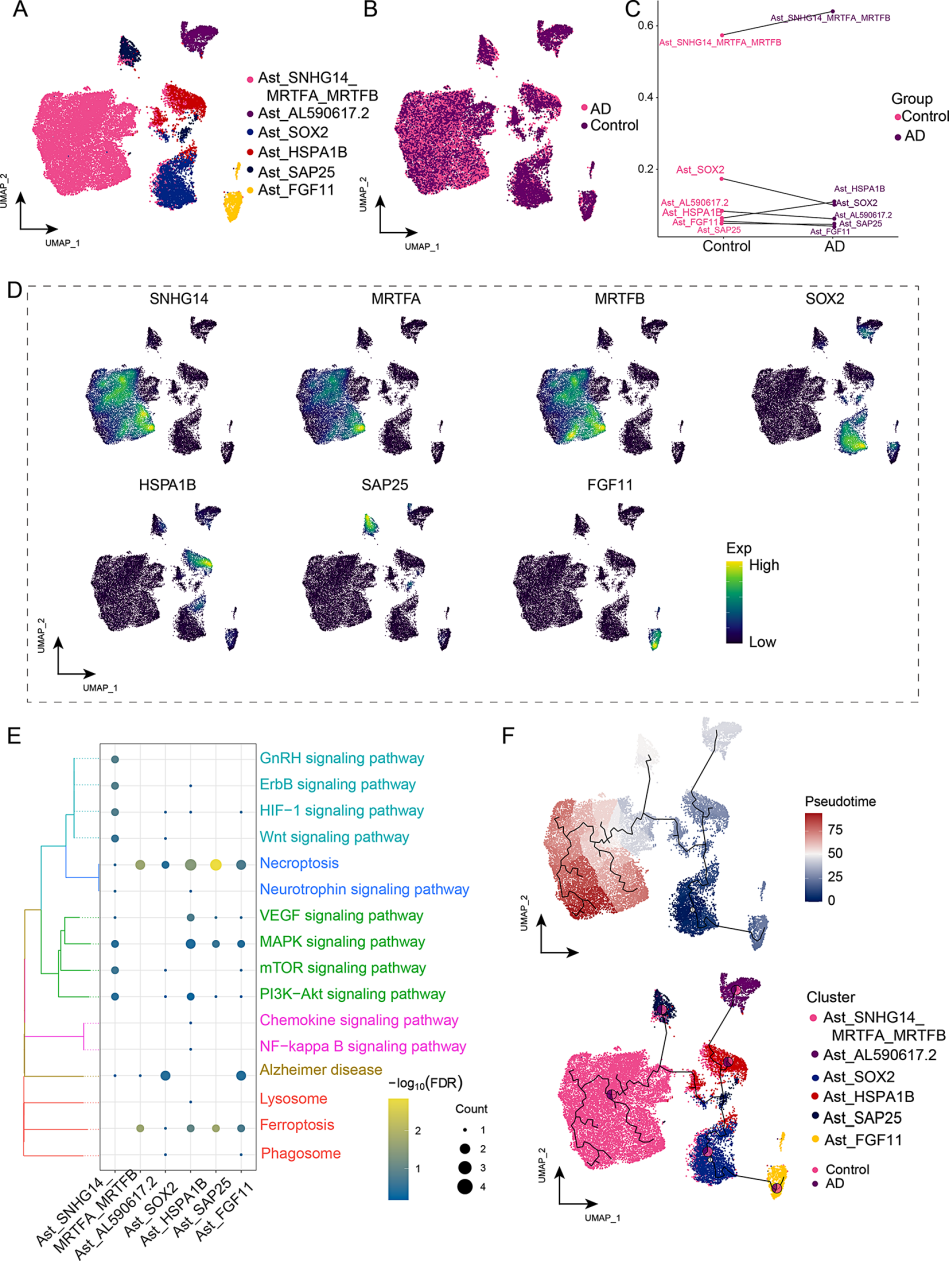
Identification of Alzheimer’s disease (AD)-associated astrocyte (Ast) clusters. **(A)** The single-nucleus atlas maps of the Ast cell clusters. **(B)** Single-nucleus map showing AD and control groups in the Ast subcluster. **(C)** Cell ecological differences of Ast clusters in different groups. **(D)** The marker genes of the Ast subcluster were plotted in the single-nucleus map. **(E)** Biological pathways significantly involved in the Ast cluster. **(F)** Single-nucleus mapping of the evolutionary trajectory of the Ast subpopulation in AD. The pie chart shows the proportion of different groups in the subpopulation. Ast, astrocytes; UMAP, uniform manifold approximation and projection

Astrocytes play a dual role in AD pathophysiology, exerting either neuroprotective or detrimental effects [40]. Astrocytes were identified in six cell subclusters (Fig. 6A), which were mapped to the AD and control groups (Fig. 6B). We also found that Ast_SOX2 was less abundant in the AD group, whereas the Ast*_*SNHG14*_*MRTFA*_*MRTFB subcluster exhibited the highest proportional increase in the AD group (Fig. 6C). We analyzed the expression of markers (*SNHG14, MRTFA, MRTFB, SOX2, HSPA1B, SAP25*, and *FGF11*) and found that lncRNA-SNHG14 was co-expressed with MRTFA and MRTFB to a certain extent (Fig. 6D). Enrichment analysis of the marker genes revealed significant enrichment in the MAPK, mammalian target of rapamycin (mTOR), phosphatidylinositide 3-kinases protein kinase B (PI3K-AKT) signaling pathways and necroptosis (Fig. 6E, Supplemental Data Table S7). The accumulation of amyloid Aβ can initiate an abnormal apoptotic cascade reaction, leading to abnormal loss of neurons [41]. The developmental trajectory of Ast revealed that the Ast*_*SOX2 subcluster was most abundant in the initial developmental stage of Ast developmental trajectory, ultimately progressing to Ast_FGF11, Ast_HSPA1B, Ast_SAP25, and Ast*_*SNHG14*_*MRTFA*_*MRTFB (Fig. 6F). These results indicate that the Ast_SNHG14_MRTFA_MRTFB subcluster is elevated in individuals with AD, which may trigger abnormal neuronal death.

### Oligodendrocyte subclusters participate in myelin formation in AD through endocytosis

**Fig. 7:**
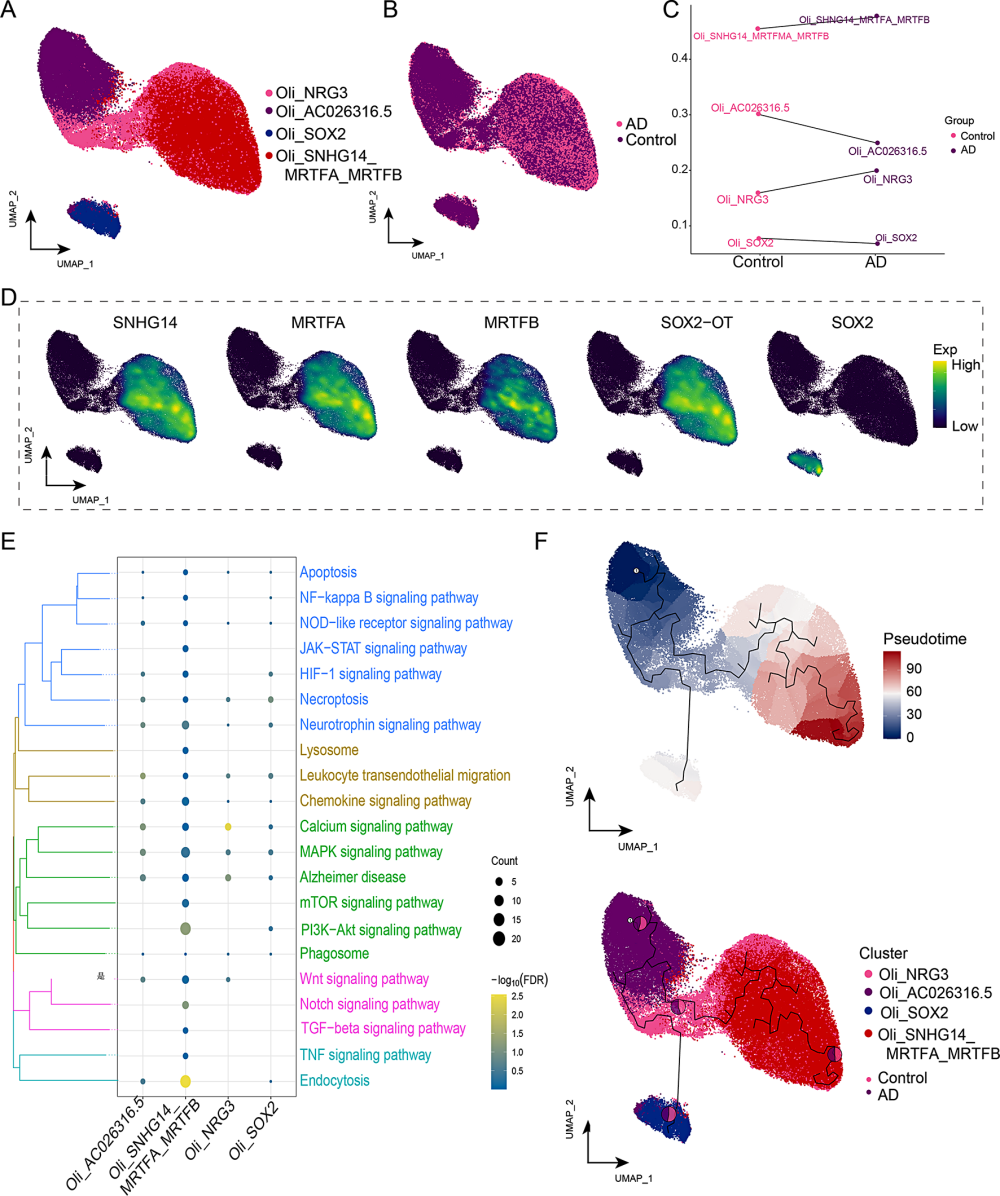
Identification of oligodendrocyte (Oli) clusters in Alzheimer’s disease (AD). **(A)** Single-nucleus map of Oli clusters. **(B)** Single-nucleus plot showing the distribution of AD and control groups in the Oli cluster. **(C)** Differences in the abundance of Oli clusters in different subgroups. **(D)** Expression of *lncRNA-SNHG14, MRTFA, MRTFB, SOX2-OT*, and *SOX2* as marker genes in the Oli subcluster. **(E)** Biological pathways of the Oli subclusters. **(F)** Single-nucleus mapping of Oli progression trajectories and pseudo-time values. The pie chart shows the proportion of different groups in the cluster. Oli, oligodendrocyte; UMAP, uniform manifold approximation and projection

The activated state of oligodendrocytes is strongly associated with AD [42]. After further clustering of the Oli, four distinct subclusters were identified (Fig. 7A) and mapped to different samples (Fig. 7B). The AD group showed an increase in the abundance of Oli*_*SNHG14*_*MRTFA*_*MRTFB cells, while the abundance of Oli_SOX2 decreased (Fig. 7C). Next, we input some marker genes into a single-nucleus map (*SNHG14, MRTFA, MRTFB, SOX2-OT, SOX2*) (Fig. 7D). The Oli subclusters were significantly involved in endocytosis, PI3K-AKT and Notch signaling pathways (Fig. 7E, Supplemental Data Table S8)Alterations in the quantity or function of oligodendrocytes and their precursors can impact the stability of myelin [43]. These pathways are already regulated in oligodendrocyte progenitors [44]; [45]. Through pseudo-time analysis, we discovered that Oli_SNHG14_MRTFA_MRTFB was abundant at the end of Oli developmental trajectory (Fig. 7F). These results indicate that Oli*_*SNHG14*_*MRTFA*_*MRTFB might regulate endocytosis and the Notch pathway to affect myelin formation.

Our study revealed the presence of subclusters co-expressed with lncRNA-SNHG14, MRTFA, and MRTFB in the above cell types. These subclusters were consistently increased in relation to the AD developmental stage, leading to Aβ aggregation, neuronal damage, and degeneration.

### Signature exploration of oligodendrocyte progenitor cell subclusters in AD

**Fig. 8:**
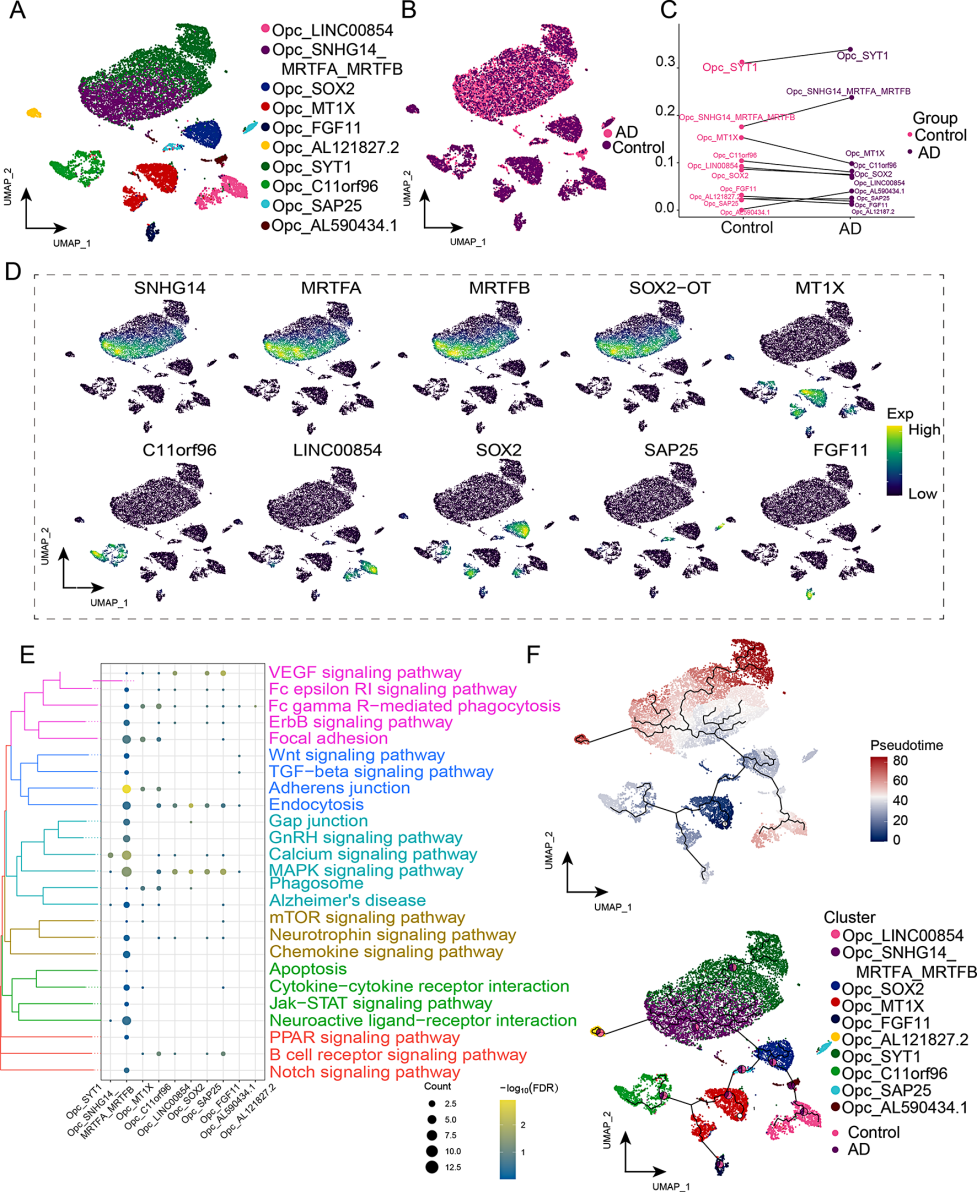
Identification of AD-associated oligodendroglial progenitor cell (Opc) clusters. **(A)** Single-nucleus maps of Opc clusters. **(B)** Single-nucleus plot showing the distribution of AD and control groups in the Opc subcluster. **(C)** Cell ecological differences of Opc subclusters in different groups. **(D)** Mapping of markers in Opc subclusters to single-nucleus maps. **(E)** Enrichment of biological pathways in Opc clusters. **(F)** Single-nucleus map of the evolutionary trajectory of Opc subpopulations in AD. The pie chart shows the proportions of different groups. AD, Alzheimer’s disease; Opc, oligodendrocyte progenitor; UMAP, uniform manifold approximation and projection

We successfully clustered oligodendrocyte progenitor cells into ten subclusters (Fig. 8A) and analyzed their distribution in the control and AD groups (Fig. 8B). Notably, we identified a subcluster that co-expressed lncRNA-SNHG14, MRTFA, and MRTFB and that exhibited increased abundance in the AD group. Additionally, we observed a decrease in Opc*_*SOX2 expression in the AD group (Fig. 8C). We then mapped the subcluster marker genes (*SNHG14, MRTFA, MRTFB, SOX2-OT, MT1X, C11orf96, LINC00854, SOX2, SAP25, FGF11*) to the single-nucleus plot (Fig. 8D). According to the enrichment analysis, Opc was enriched in various biological pathways, including adherens junction, endocytosis, focal adhesion kinase, calcium and MAPK signaling pathways (Fig. 8E, Supplemental Data Table S9). Using pseudo-time analysis, we predicted that cellular developmental trajectory began with Opc_MT1X and progressed to Opc*_*FGF11, Opc*_*C11orf96, Opc_SAP25, Opc_SOX2, Opc_LINC00854, and Opc_SNHG14_MRTFA_MRTFB (Fig. 8F). These findings suggest that the Opc subclusters contribute primarily to myelination in AD and that Opc_SNHG14_MRTFA_MRTFB may impact myelin formation and regeneration in AD development. Based on our findings, we hypothesized that the co-expressed subclusters of LncRNA-SNHG14, MRTFA, and MRTFB may create a pathogenic microenvironment in AD.

### Cell-cell communication in the AD microenvironment

**Fig. 9:**
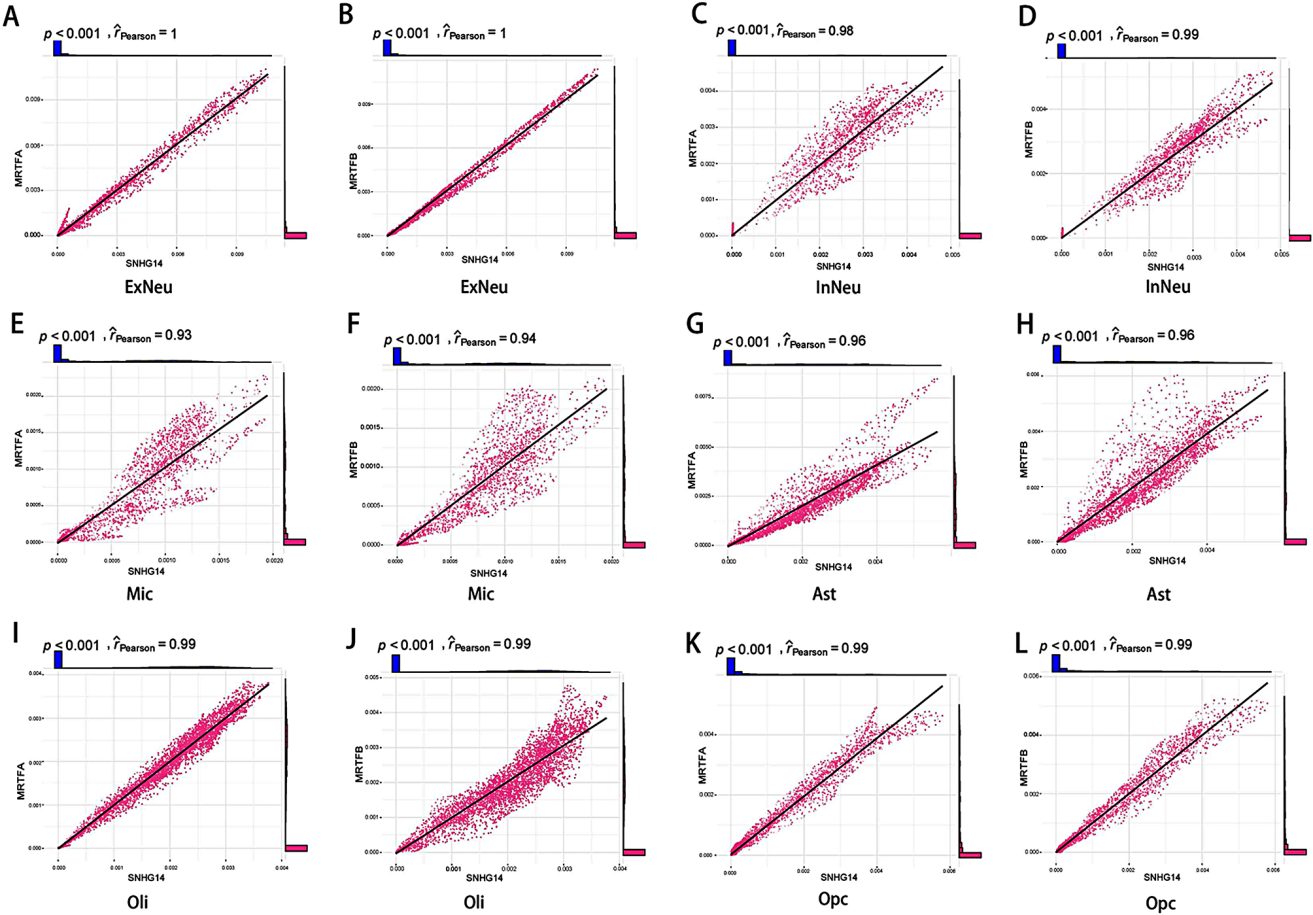
PlotCor of SNHG14 and MRTFA, MRTFB in six cell type. (A) PlotCor of SNHG14 andMRTFA in ExNeu. (B) PlotCor of SNHG14 and MRTFB in ExNeu. (C) PlotCor of SNHG14 and MRTFA in inNeu. (D) PlotCor of SNHG14 and MRTFB in lnNeu. (E) PlotCor of SNHG14 and MRTFA in Mic. (F) PlotCor of SNHG14 and MRTFB in Mic. (G)PlotCor of SNHG14 and MRTFA in Ast. (H) PlotCor of SNHG14 and MRTFB in Ast. (I) PlotCorof SNHG14 and MRTFA in Oli. (J) PlotCor of SNHG14 and MRTFB in Oli. (K) PlotCor of SNHG14 and MRTFA in Opc. (L) PlotCor of SNHG14 and MRTFB in Opc. P value < 0.001, R pearson represent relevance, the higher the value, the higher the correlation

**Fig. 10:**
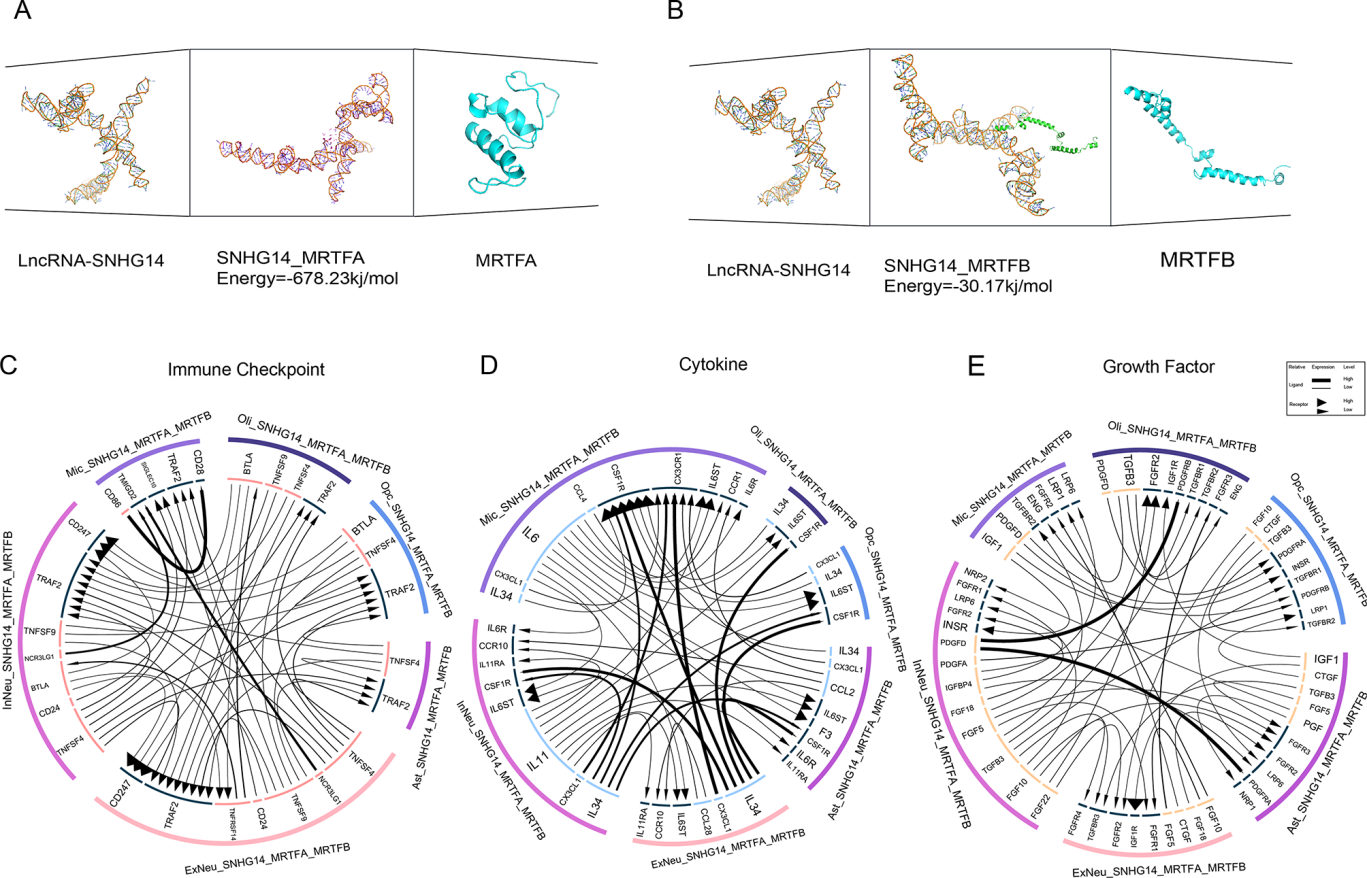
Verification of the interactions between lncRNA-SNHG14, MRTFA, and MRTFB. **(A)** Molecular docking model demonstrating the binding site for lncRNA-SNHG14 and MRTFA. **(B)** Molecular docking model demonstrating the binding site for lncRNA-SNHG14 and MRTFB. The smaller the value, the greater the binding ability. **(C)** Immune checkpoint modules for intracellular and intercellular communication in cellular communication networks. **(D)** Intracellular and intercellular communication cytokine modules in cellular communication networks. **(E)** Intracellular and intercellular communication growth factor modules in cellular communication networks

We found subclusters co-expressing lncRNA-SNHG14, MRTFA, and MRTFB in all six cell types, revealing a positive correlation between lncRNA-SNHG14, MRTFA, and MRTFB expression (Fig. 9). Based on molecular docking, lncRNA-SNHG14 was confirmed to bind MRTFA and MRTFB **(**Fig. 10A, B**)**. We evaluated the cellular interactions of the three modules, and immune checkpoint modules (Fig. 10C) revealed interactions between TNFSF9 and TRAF2 that occurred in both neurons and glial cells in AD and showed that TNFSF9 may bind TRAF2 in AD [46]. The cytokine module (Fig. 10D) suggested that excitatory and inhibitory neurons might influence the other four cell types through the IL-34-CSF1R pathway, leading to a decrease in the ability of neurons to take up pathological forms of Aβ [47]. The growth factor module (Fig. 10E) involves PDGFD-PDGFRA activation and PDGFD-PDGFRB signaling, which regulates various functions of the central nervous system [48]. The cell communication results presented here indicate a strong correlation between neurons and glial cells and that subclusters expressing lncRNA-SNHG14, MRTFA, and MRTFB may be associated with poor prognosis of AD.

## Discussion

This analysis was based on a large sample set that allowed us to identify six cell types, including ExNeu, InNeu, Mic, Ast, Oli, and Opc. We analyzed cell subclusters and found significant differences in AD and control groups. Additionally, we explored cell developmental trajectories and cell-cell interactions. This study offers insights into the molecular mechanisms underlying AD and provides a foundation for AD theory.

We discovered genes that were not present in the two datasets and identified a subcluster of co-expressed lncRNA-SNHG14, MRTFA, and MRTFB. The abundance of this subcluster gradually increased during AD development. Previous studies found that SNHG14, as a lncRNA, plays an important role in neurological disease [49], whereas SNHG14 knockdown reduces neurotoxicity [50]. We found that ExNeu_SNHG14_MRTFA_MRTFB and InNeu_SNHG14_MRTFA_MRTFB were significantly enriched in calcium signaling, neuroactive ligand-receptor interaction, and MAPK signaling pathways. Indeed, neurons can cause dysregulation of Ca^2+^ homeostasis to promote the formation of Aβ plaques and neurofibrillary tangles [34]. Neuroactive ligand-receptor interactions are associated with cognition, learning, and memory [35]. Additionally, the MAPK pathway participates in AD progression [37]. Hence, the neuronal SNHG14_MRTFA_MRTFB subcluster might elicit destructive processes associated with the neuronal damage or degeneration observed in AD.

Our results also indicated that Mic_SNHG14_MRTFA_MRTFB was enriched in various immune-related pathways, including B cell receptor, T cell receptor, chemokine signaling, and Fc R-mediated phagocytosis pathways. Moreover, this cell subcluster was found to be abundant late in the microglial developmental trajectory. LncRNA-SNHG14 reportedly promotes microglial activation in ischemic stroke, leading to the release of myriad inflammatory cytokines and promoting neuronal damage [51]. Microglia are responsible for secreting chemokines in AD [52]. As microglia age, they promote the infiltration of peripheral T-cells, which can lead to brain damage [53]. In fact, blocking microglia or cytotoxic T cells prevents tau-mediated neurodegeneration [54]. Taken together, these results suggest that microglia mediate inflammatory responses in late AD and promote AD progression.

Astrocytes play a significant role in protecting the surrounding brain tissue from Aβ species and secreting cytokines and neurotoxic products [55]. Our study showed that astrocytes were mainly enriched in MAPK, mTOR, and PI3K-Akt signaling pathways and necroptosis, which have been shown to trigger abnormal apoptotic cascades in AD and lead to abnormal neuronal loss [41]. Moreover, lncRNA-SNHG14 participates in the inflammatory response of astrocytes in AD [56]. Hence, we suggest that the Ast_SNHG14_MRTFA_MRTFB subcluster is responsible for the pathological progression of AD and that it plays an important role in regulating apoptosis during late cell development and inducing neuronal degeneration.

Changes in the number or function of oligodendrocytes and their precursors can impact myelin sheath integrity [43]. Recent studies have shown that the Notch signaling pathway participates in demyelination and remyelination in the central nervous system [57]. Moreover, oligodendrocytes expressing the low-density lipoprotein receptor endocytose cholesterol to maintain adult myelination [44]. Our analysis of the Oli and Opc clusters revealed subclusters that co-expressed lncRNA-SNHG14, MRTFA, and MRTFB. Surprisingly, they were also in the terminal stage of the cell development trajectory. Therefore, this subcluster may impact myelination maintenance in AD through endocytosis. However, a role for lncRNA-SNHG14 in oligodendrocytes has not yet been reported, and further research is required to understand the significance of this finding.

The identification of various cell subclusters co-expressing lncRNA-SNHG14, MRTFA, and MRTFB is consistent with the observation that MKL1/MRTFA is abnormally aggregated in human AD postmortem brain tissues [58], indicating that the disruption of MKL1/MRTFA function may contribute to the progression of AD pathology [59]. Indeed, lncRNA-SNHG14, MRTFA, and MRTFB play crucial roles in neurological diseases as activators of sero-response factors that regulate related target genes [60].

Analysis of cell-cell communication pathways revealed potential links that may regulate AD pathology. Specifically, we identified interactions between TNFSF9-TRAF2, IL-34-CSF1R, PDGFD-PDGFRA, and PDGFD-PDGFRB. Research has indicated that TNFSF9 expression is not properly regulated in late-onset AD [61]. Meanwhile, the TNF-α/TNF-R signaling pathway involves complex interactions between multiple proteins, including TNF-receptor-associated factor-2 (TRAF-2) [46]. CSF-1R and its ligand IL-34 also play crucial roles in the regulation of microglial and neuronal lineages in the brain [62]. In late-onset AD, PDGF-BB affects downstream signaling for PDGFR-β pericyte dysfunction [63, 64]. Moreover, microglia impact the regulation of other glial cells and immunity, thereby participating in the pathological processes of AD [65]. Taken together, these data suggest that the six cell types function as a complex interconnected network and may form a pathogenic microenvironment. As AD progresses, SNHG14, MRTFA, and MRTFB may exist in a disease-associated microenvironment and play an important role in promoting AD development.

We also observed a decrease in the levels of ExNeu_MTRNR2L8 and InNeu_MTRNR2L8 in AD. Previous studies have indicated that MTRNR2L8 may serve as a diagnostic biomarker and therapeutic target for stroke [66]. However, there are currently no reports related to these findings in AD. Hence, a potential role for the downregulation of MTRNR2L8 in AD progression warrants further investigation. SOX2, a key transcription factor in neurogenesis regulation [67], showed a significant decrease in expression in astrocytes, oligodendrocytes, and oligodendrocyte progenitors. Previously, The expression of SOX2 was inversely proportional to the severity of the transgenic AD mouse model [68]. We also found that SOX2-OT—a non-coding RNA—exists in oligodendrocytes and oligodendrocyte progenitors. SOX2-OT’s introns contain the single-exon SOX2 [69]. An unbiased study suggested that SOX2-OT might serve as a biomarker of neurodegeneration [70], and, based on our results, it may play an important role in the development of an AD microenvironment.

This study had certain limitations. Our investigation focused solely on tissue from a specific area of the brain, the prefrontal cortex, rather than examining the brain as a whole. Therefore, it will be essential to conduct comprehensive studies involving various brain regions to confirm and validate these findings. Second, this study was focused on bioinformatics analyses. The precise mechanisms underlying our findings must be confirmed through relevant molecular and cellular studies.

## Conclusion

Our study provides a comprehensive understanding of the single-nucleus signature of AD based on six distinct cell types. In our analyses, we identified a subcluster co-expressing lncRNA-SNHG14, MRTFA, and MRTFB in these six cell types. The abundance of these subclusters increased in AD, suggesting a possible association with AD microenvironment. However, signaling pathways, such as MAPK, calcium signaling, immune-related, apoptosis, and endocytosis pathways, varied across subclusters. In particular, lncRNA-SNHG14 was found to regulate MRTFA and MRTFB. Further investigations into the distribution and localization of MTRNR2L8, SOX2, and SOX2-OT in different cell types are warranted.

### Electronic supplementary material

Below is the link to the electronic supplementary material.

Supplementary Material 1

Supplementary Material 2

Supplementary Material 3

Supplementary Material 4

Supplementary Material 5

Supplementary Material 6

Supplementary Material 7

Supplementary Material 8

Supplementary Material 9

Supplementary Material 10

## Data Availability

Data was download from the public database online at the GEO database under accession numbers GSE157827 and GSE174367.

## Abbreviations

AD: Alzheimer’s disease
BP: Biological process
CC: Cellular component
DEG: Different expression gene
ExNeu: Excitatory neuron
GEO: Gene Expression Omnibus
InNeu: Inhibitory neuron
KEGG: Kyoto Encyclopedia of Genes and Genomes
lncRNA: Long noncoding RNA
MAPK: mitogen-activated protein kinase
Mic: Microglia
MF: Molecular function
MTRNR2L8: MT-RNR2-like 8
MRTFA: Myocardin-related transcription factor A
MRTFB: Myocardin-related transcription factor B
NFTs: Neurofibrillary tangles
Opc: Oligodendrocyte progenitor
Oli: Oligodendrocyte
snRNA: seq-Single-nucleus ribonucleic acid sequencing
SOX2: SRY-box transcription factor 2
UMAP: Uniform manifold approximation and projection
