# Supplementary material for: Single-nucleus analysis reveals microenvironment-specific neuron and glial cell enrichment in Alzheimer’s disease
Source: BMC Genomics. 2024 May 28;25:526. doi: 10.1186/s12864-024-10447-3 (PMC11134750; doi:10.1186/s12864-024-10447-3)
Supplement: Supplementary file 10 — Supplementary Material 10 [file 12864_2024_10447_MOESM10_ESM.pdf]

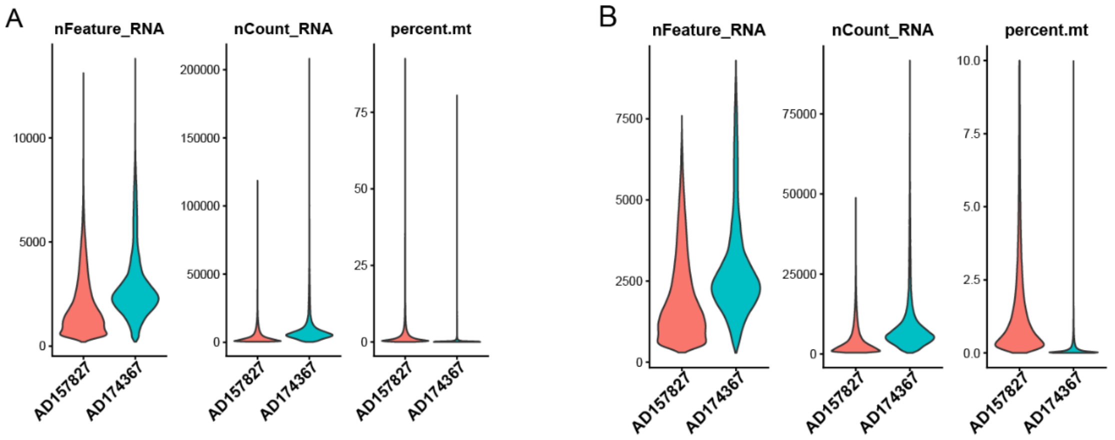

Supplemental Figure S1: Number of genes before and after quality control. **(A)** Before quality control. **(B)** After quality control. The first plot is a violin plot of the number of genes detected for all cells in two datasets, and the ordinate is the number of genes contained in each cell. The second plot shows the violin plot of the number of mid-gene sequences in all cells of the sample, with the abscissa for two datasets and the ordinate for the number of genes per cell. The third plot shows the violin plot of the proportion of mitochondria in all cells of two datasets and the ordinate for the proportion of mitochondria in each cell.

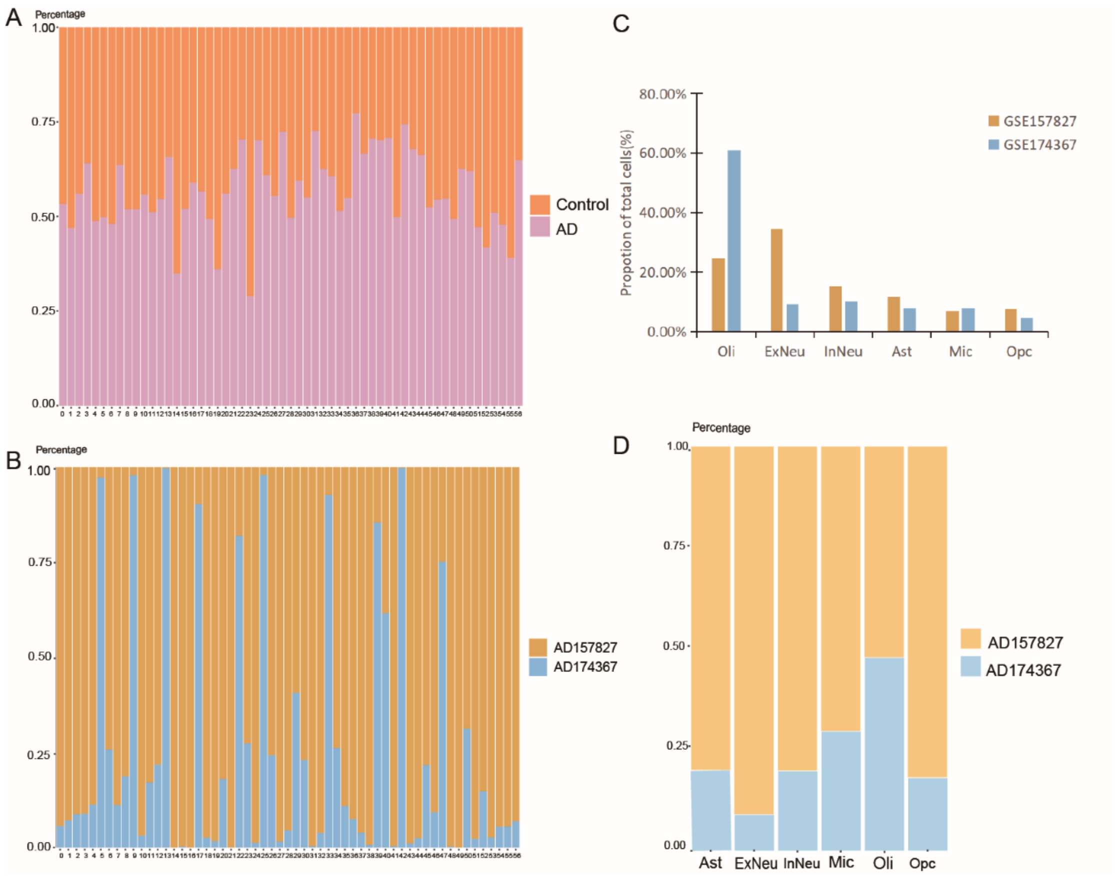

Supplemental Figure S2: Changes in the abundance of total cells in different clusters in different group. **(A)** Changes in the abundance of total cells of different clusters in

patients with AD and control. **(B)** Changes in the abundance of total cells of different clusters in GSE157827 and GSE174367. **(C)** Proportion of total cell numbers in GSE157827 and GSE174367. **(D)** Changes in the abundance of total cells of different cell type inGSE157827 and GSE174367.
